# The Resistance to Host Antimicrobial Peptides in Infections Caused by Daptomycin-Resistant *Staphylococcus aureus*

**DOI:** 10.3390/antibiotics10020096

**Published:** 2021-01-20

**Authors:** Md Saruar Bhuiyan, Jhih-Hang Jiang, Xenia Kostoulias, Ravali Theegala, Graham J. Lieschke, Anton Y. Peleg

**Affiliations:** 1 Infection and Immunity Program, Monash Biomedicine Discovery Institute, Department of Microbiology, Monash University, Clayton, VIC 3800, Australia; saruar.bhuiyan@path.utah.edu (M.S.B.); jhih-hang.jiang@monash.edu (J.-H.J.); xenia.kostoulias@monash.edu (X.K.); rthe0003@student.monash.edu (R.T.); 2 Australian Regenerative Medicine Institute, Monash University, Clayton, VIC 3800, Australia; graham.lieschke@monash.edu; 3 Department of Infectious Diseases, The Alfred Hospital and Central Clinical School, Monash University, Melbourne, VIC 3004, Australia

**Keywords:** *S. aureus*, daptomycin, antimicrobial peptides, persistent infection

## Abstract

Daptomycin is an important antibiotic for the treatment of infections caused by *Staphylococcus aureus*. The emergence of daptomycin resistance in *S. aureus* is associated with treatment failure and persistent infections with poor clinical outcomes. Here, we investigated host innate immune responses against clinically derived, daptomycin-resistant (DAP-R) and -susceptible *S. aureus* paired isolates using a zebrafish infection model. We showed that the control of DAP-R *S. aureus* infections was attenuated in vivo due to cross-resistance to host cationic antimicrobial peptides. These data provide mechanistic understanding into persistent infections caused by DAP-R *S. aureus* and provide crucial insights into the adaptive evolution of this troublesome pathogen.

## 1. Introduction

*Staphylococcus aureus* continues to be one of the most important human bacterial pathogens, with the capacity to cause a broad range of infections, including *S. aureus* bacteremia (SAB) [1,2,3]. Treatments of SAB caused by methicillin-resistant *S. aureus* (MRSA) have increasingly relied on last-line antibiotics, particularly vancomycin and daptomycin [4,5,6]. Daptomycin is a cyclic lipopeptide that targets bacterial membranes to execute bactericidal effects [7,8]. The mechanisms of daptomycin actions are proposed to be similar to host cationic antimicrobial peptides (CAMPs) [7,8]. Notably, the emergence of daptomycin resistance has been associated with persistent and complicated staphylococcal infections in patients [4,9]. Recent studies have also shown that clinically derived daptomycin-resistant (DAP-R) isolates caused persistent infections in *Galleria mellonella* infections and murine septicemia models [10,11,12,13]. The correlation between daptomycin resistance and prolonged bacterial survival in the infected host demands further investigation.

CAMPs are important constituents of the innate immune system in mammals that influence multifaceted biological processes [14,15]. CAMPs process antimicrobial activities against microorganisms, including bacterial pathogens. Most CAMPs are cationic in nature with positive charges ranging from +2 to +9, which are thought to target negatively charged bacterial membranes via electrostatic interactions [14,15]. Perturbations of bacterial membranes are considered to be the main bactericidal mechanisms of CAMPs [14,15]. In addition to antimicrobial properties, CAMPs can also prompt the adaptive immune system via the chemoattraction of immature dendritic cells and memory T cells [16]. CAMPs have been shown to be important innate host defenses against bacterial infection, including *S. aureus* [17,18,19]. Of note, cross-resistance to host CAMPs has been reported in clinically derived DAP-R *S. aureus* isolates in in vitro studies [20,21]. However, it is unclear that the cross-resistance to CAMPs contributes to persistent infections caused by DAP-R *S. aureus* strains in a whole-animal infection model.

The use of the vertebrate model system *Danio rerio* (zebrafish) has recently revealed essential aspects of the interactions between host and pathogens [22,23]. Zebrafish share a remarkably similar immune system to humans, including innate and adaptive immunity, and have been used to study host immune responses against bacterial pathogens [23]. For instance, efforts in zebrafish have shown the evolutionary conserved role for nerve growth factor β and its receptor tyrosine kinase TrkA signaling in pathogen-specific host immunity against *S. aureus* [23]. Our recent study shows that *S. aureus* can evade neutrophil chemotaxis in zebrafish by reducing bacterial membrane phosphatidylglycerol through point mutations in the phospholipid biosynthesis gene *cls2*, encoding cardiolipin synthase [22].

In the present study, we investigated the impact of DAP-R *S. aureus* infection on host antimicrobial peptide responses in vivo, which provides insights into persistent staphylococcal infections.

## 2. Results

**Figure 1:**
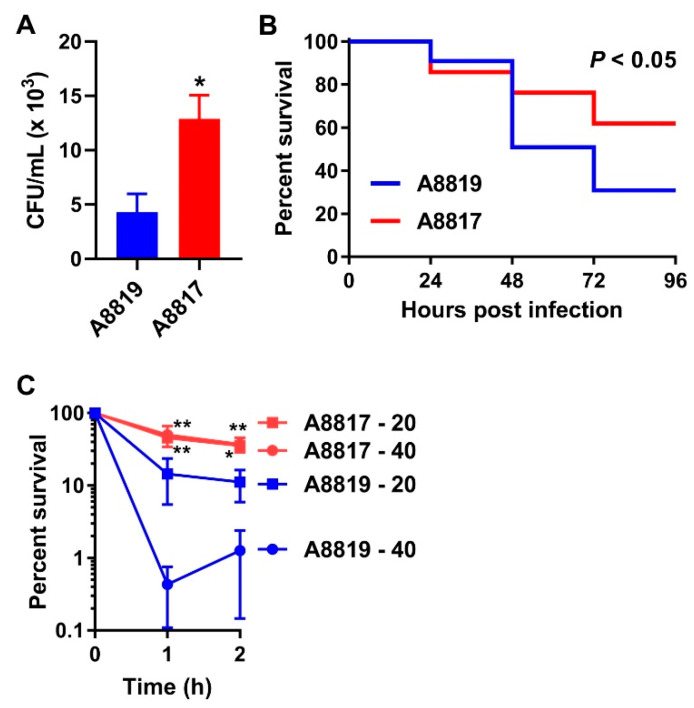
Impact of daptomycin resistance on in vivo virulence and host immune responses. (**A**) Survival of the clinical DAP-R isolate A8817 and its parental DAP-S strain A8819 over 3 h in human blood (*n* = 4; * *p* < 0.05, Mann–Whitney test). (**B**) Survival of zebrafish following bloodstream infection with live *S. aureus* isolates (*n* = 30 embryos, three biological replicates; *P* value is a comparison of A8819 and A8817 by log-rank test). (**C**) Survival of the clinical DAP-R isolate A8817 and its parental DAP-S strain A8819 over 2 h in the presence of 20 μg/mL and 40 μg/mL hNP-1 (*n* = 3; for A8817 versus A8819, * *p* < 0.05 and ** *p* < 0.01 at indicated time points for same dosages, Student’s *t*-tests). Error bars represent the mean ± SEM. hNP-1: human neutrophil peptide 1.

We collected *S. aureus* isolates from a patient with a complicated and persistent bloodstream infection that was treated with daptomycin but failed therapy, including a DAP-S parental strain, A8819, and its corresponding DAP-R daughter strain, A8817, that emerged after clinical failure [9]. To investigate the relationship between daptomycin resistance and persistent infections, we first measured the capacity of human whole blood to kill the paired DAP-S and DAP-R isolates ex vivo. DAP-R strain A8817 was significantly resistant to the killing of innate immune responses in blood compared to its parental DAP-S strain A8819 (Figure 1A). To further assess the virulence of these strains in vivo, we utilized the vertebrate zebrafish (*Danio rerio*) model system [23]. DAP-S strain A8819 caused lethal disease in zebrafish following a bloodstream infection, whilst its daughter strain, A8817, was significantly attenuated for virulence (Figure 1B). These data were consistent with what we and others have previously shown with multiple clinical, daptomycin-exposed pairs in a murine septicemia model [10,11,12].

**Figure 2:**
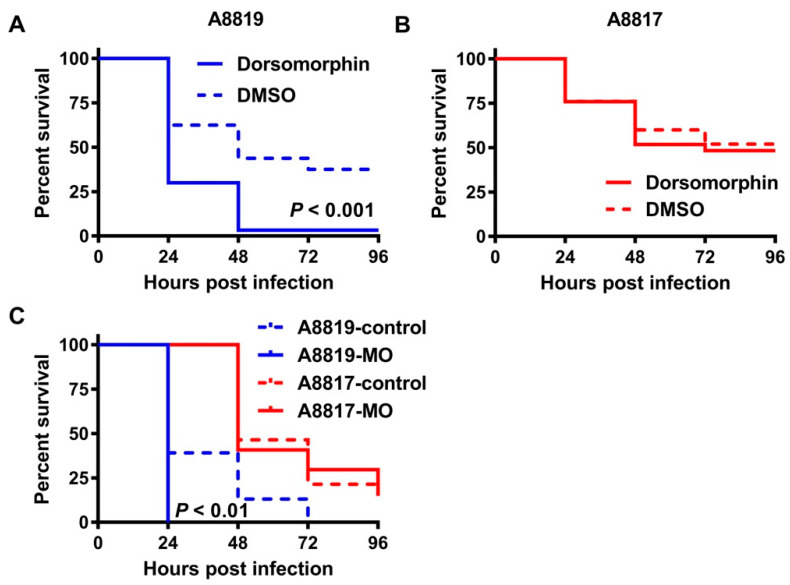
Impact of daptomycin resistance on infection control by antimicrobial peptides in vivo. Survival of zebrafish infected with (**A**) A8819 and (**B**) A8817 after treatments with dorsomorphin or dimethyl sulfoxide (DMSO, solvent for dorsomorphin). (**C**) Survival of zebrafish infected with A8819 and A8817 after injections with hepcidin morpholino oligos (MO) or scrambled morpholino oligos (control). (*n* = 25 embryos, three biological replicates; *p* value is a comparison of dorsomorphin and DMSO for (**A**), hepcidin morpholino and the negative control for A8819 for (**C**) by log-rank test).

We hypothesized that resistance to host CAMPs may contribute to persistent infections caused by DAP-R *S. aureus*. We first assessed this in vitro using human neutrophil peptide 1 (hNP-1). We showed that hNP-1 was bactericidal against DAP-S isolate A8819, with the most profound effects observed at 40 μg/mL over 2 h (Figure 1C). However, hNP-1 had little effect on the survival of the DAP-R isolate A8817 (Figure 1C). To determine the impact of this cross-resistance to daptomycin and CAMPs in vivo, zebrafish were incubated in dorsomorphin, which inhibits a key antimicrobial peptide known as hepcidin [24,25], prior to bacterial infection and for the duration of the experiment. We expected that if CAMPs were important in vivo, dorsomorphin would lead to augmented virulence of A8819, but would have no effect on A8817 infection due to its resistance and independence of CAMPs. Treatment with dorsomorphin significantly enhanced the virulence of A8819 (Figure 2A). This treatment had no effect on DAP-R A8817 infection (Figure 2B). To support these findings further, we also silenced hepcidin mRNA using a targeted morpholino. Injection of the hepcidin morpholino significantly increased the virulence of A8819 compared to the treatment with a standard negative control morpholino, whereas the virulence of the DAP-R strain A8817 remained unaffected by the knockdown of hepcidin (Figure 2C).

## 3. Discussion

Emerging resistance to last-line anti-staphylococcal agents has raised concerns regarding therapeutic options. In particular, infections caused by DAP-R *S. aureus* are often persistent, complicated and difficult to eradicate [4,9]. Here, we report that *S. aureus* became equipped with the ability to evade host innate immune responses during the evolution of daptomycin resistance. This immune evasion involved cross-resistance to important host antimicrobials, CAMPs. Together, the ability to circumvent crucial innate defenses provides important insights into the complex and persistent infections observed, yet unexplained, with DAP-R *S. aureus* infections in patients.

CAMPs are significant native components of innate host defense and provide protection against infections caused by bacterial pathogens, including *S. aureus*, Group A *Streptococcus*, and *Salmonella typhimurium* [17,18,26]. Resistance to CAMPs in vivo has been shown to promote persistent bacterial infections with Group A *Streptococcus*, with strains resistant to cathelicidin causing more severe and prolonged skin infections in a murine model [17]. However, the impact of CAMP resistance in vivo has not been shown with *S. aureus* thus far. Similar to previous studies [20,21], our current research showed that daptomycin resistance in *S. aureus* led to cross-resistance to host CAMPs in vitro. However, here we also showed the impact of daptomycin resistance on disease and CAMP sensitivity and control in vivo. We showed that CAMPs were important in controlling *S. aureus* infection, a phenotype dependent on CAMP sensitivity. Infection with DAP-R A8817 was unaffected by the presence or absence of the zebrafish CAMP hepcidin, whereas infection with the paired DAP-S strain A8819 caused greater mortality when hepcidin was inhibited. Future analyses are still required to investigate the mechanisms behind the cross-resistance to CAMPs and the reduced virulence of the DAP-R strain A8817 in animal infection models. In summary, the DAP-R strain was disarming the host of its most effective first-line immune defenders, providing important insights into the stealthy behavior of pathogenic *S. aureus*.

## 4. Materials and Methods

### 4.1. Culture of Bacterial Strains and Human Neutrophil Peptide 1 (hNP-1) Killing Assay

Clinically derived DAP-S *S. aureus* isolates A8819 and its DAP-R daughter strain A8817 were used as previously described [9]. *S. aureus* cells were cultured at 37 °C with constant shaking in Bacto^TM^ Brain Heart Infusion broth (BHI) (BD, Franklin Lakes, NJ, USA). For bactericidal activity of hNP-1, *S. aureus* cells were diluted into 10 mM KH_2_PO_4_, pH 7.4, containing 1% BHI broth and hNP-1 (20 μg/mL or 40 μg/mL) (Peptide Institute, INC, Osaka, Japan) to achieve a final inoculum of 10^6^ CFU/mL and then incubated at 37 °C for 2 h. At the indicated time point, viable cells were quantified by plating the cell suspension on BHI agar plates after serial dilutions.

### 4.2. Ex Vivo Human Whole Blood Killing Assay

Bacterial suspensions in 50 μL PBS were mixed with 50 μL human blood to achieve final bacterial density at 2 × 10^4^ CFU/mL in a 96-well plate. The plate was incubated at 37 °C for 3 h under continuous shaking. The number of bacterial CFU was determined after incubation by plating serial 10-fold dilutions.

### 4.3. Zebrafish Infection, Leukocyte Enumeration and Survival Analyses

Wild-type Tübingen and Tg(*lyz*:DsRed)^nz50^ zebrafish embryos were maintained in the Monash University AquaCore facility according to standard protocols [22]. Zebrafish embryos (48 h post-fertilization, hpf) were injected with *S. aureus* (1000 CFU/embryo) in common cardinal vein for a bloodstream infection for survival analyses. Five embryos were homogenized immediately after infection each time and plated on BHI agar to confirm bacterial inoculums. Ten embryos per treatment were monitored daily for survival up to 96 h post-infection (hpi) and dead embryos were recorded at each time point in three independent experiments. Zebrafish work was approved by the Monash University Animal Ethics Committee (MAS/2010/18).

### 4.4. Inhibition of the Antimicrobial Peptide Hepcidin In Vivo

The major zebrafish antimicrobial peptide hepcidin [25] was inhibited using two approaches. First, chemical inhibition was performed by incubating embryos in egg water containing 40 μM dorsomorphin from 30 hpf until the conclusion of the experiment. Embryos treated with egg water containing 0.3% DMSO, which was used to solubilize dorsomorphin, were used as a control. Dorsomorphin inhibits the bone morphogenetic protein signaling pathway, which is required for producing hepcidin [24]. Second, we inhibited hepcidin expression using antisense morpholino oligomers (MO) (CACGTTAGAAAGCTTCATCTTCAGT) directed at *hamp* (Gene Tools, LLC, Eugene OR). Yolks of one-cell embryos were injected with 1 nL of 25 mM *hamp*ATG MO in distilled water per embryo. A standard negative control MO (CCTCTTACCTCAGTTACAATTTATA) was used as a negative control.

### 4.5. Statistical Analysis

Statistics were generated using GraphPad Prism version 6.0, GraphPad Software, La Jolla California USA, www.graphpad.com. Statistical tests were performed as indicated in the figure legends. *p* < 0.05 was considered significant for all analyses.

## Data Availability

Data is contained within the article.
